# Supplementary material for: Bacteria invade the brain following intracortical microelectrode implantation, inducing gut-brain axis disruption and contributing to reduced microelectrode performance
Source: Nat Commun. 2025 Feb 20;16:1829. doi: 10.1038/s41467-025-56979-4 (PMC11842729; doi:10.1038/s41467-025-56979-4)
Supplement: Supplementary file 1 — Supplementary Information [file 41467_2025_56979_MOESM1_ESM.pdf]

# Bacteria Invade the Brain Following Intracortical Microelectrode Implantation, Inducing Gut-Brain Axis Disruption and Contributing to Reduced Microelectrode Performance

**George F. Hoeferlin[1,2], Sarah E. Grabinski[3], Lindsey N. Druschel[1,2], Jonathan L. Duncan[1,2], Grace Burkhart[1], Gwendolyn R. Weagraff[2,4], Alice H. Lee[1,2], Christopher Hong[1,2], Meera Bambroo[1,2], Hannah Olivares[1,2], Tejas Bajwa[1,2], Jennifer Coleman[1], Longshun Li [1,2], William Memberg[1,5], Jennifer Sweet[5,6], Hoda Amani Hamedani[2,7], Abhinav P. Acharya[1], Ana G. Hernandez-Reynoso[8], Curtis Donskey[5,9], George Jaskiw[5,10], E. Ricky Chan[11], Andrew J. Shoffstall [1,5], A. Bolu Ajiboye[1,5], Horst A. von Recum[1,2], Liangliang Zhang[3,12,*], Jeffrey R. Capadona[1,2,*]**

1. Department of Biomedical Engineering, Case Western Reserve University, Cleveland, OH 44106, USA
2. Advanced Platform Technology Center, Louis Stokes Cleveland Department of Veterans Affairs Medical Center, Cleveland, OH 44106, USA
3. Department of Population and Quantitative Health Sciences, Case Western Reserve University, Cleveland, OH 44106, USA
4. University of Florida, Department of Biology, Gainesville, FL 32611, USA
5. Louis Stokes Cleveland Department of Veterans Affairs Medical Center, Cleveland, OH 44106, USA
6. Department of Neurological Surgery, University Hospitals Case Medical Center, 11100 Euclid Ave, Cleveland, OH, 44106, USA
7. Department of Materials Science and Engineering, Case Western Reserve University, Cleveland, OH 44106, USA
8. Department of Bioengineering, The University of Texas at Dallas, 800 W. Campbell Road, Richardson, TX 75080, USA
9. Division of Infectious Diseases & HIV Medicine in the Department of Medicine, Case Western Reserve University School of Medicine, Cleveland, OH 44106, USA
10. Department of Psychiatry, Case Western Reserve University, Cleveland, OH 44106, USA
11. Cleveland Institute for Computational Biology, Case Western Reserve University, Cleveland, OH 44106, USA
12. Case Comprehensive Cancer Center, Case Western Reserve University, Cleveland, OH 44106, USA

*Correspondence: Jeffrey R. Capadona (jrc35@case.edu) or Liangliang Zhang (lxz716@case.edu)

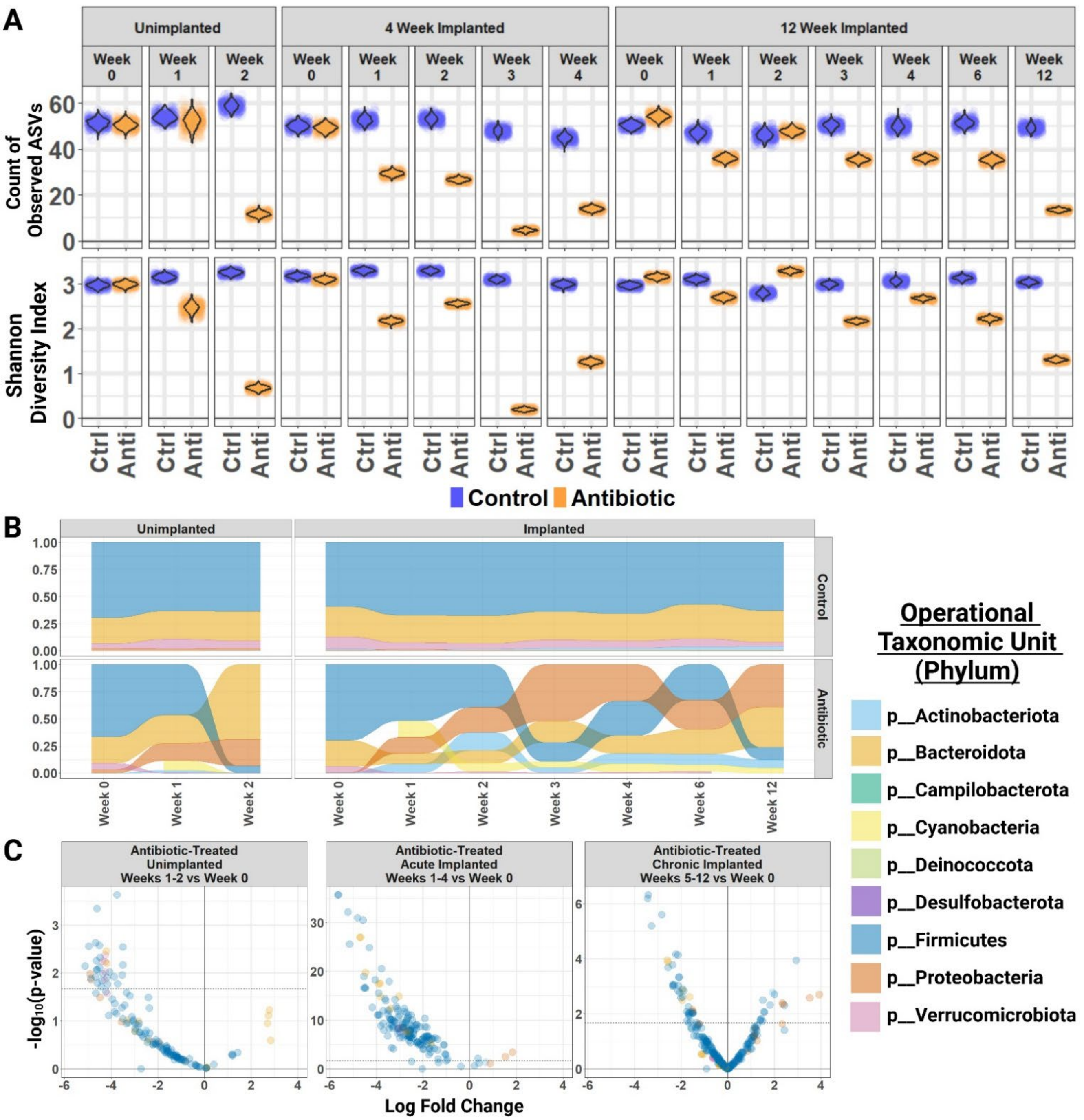


**Supplemental Figure S1: Antibiotic Treatment Significantly Impacts Gut Microbiome Composition.** Fecal data was obtained from all animals and cohorts involved in the study as a measure of gut microbiome composition. Biological replicate sample sizes as $n_{week}$: unimplanted control ($n_0$ = 5, $n_1$ = 5, $n_2$ = 5), unimplanted antibiotic ($n_0$ = 5, $n_1$ = 2, $n_2$ = 4), implanted 4-week control ($n_0$ = 6, $n_1$ = 6, $n_2$ = 6, $n_3$ = 6, $n_4$ = 6), implanted 4-week antibiotic ($n_0$ = 6, $n_1$ = 5, $n_2$ = 5, $n_3$ = 4, $n_4$ = 3), implanted 12-week control ($n_0$ = 8, $n_1$ = 4, $n_2$ = 4, $n_3$ = 7, $n_4$ = 4, $n_6$ = 7, $n_{12}$ = 7), implanted 12-week antibiotic ($n_0$ = 8, $n_1$ = 5, $n_2$ = 5, $n_3$ = 7, $n_4$ = 6, $n_6$ = 6, $n_{12}$ = 3). Unimplanted/Implanted, Control/Antibiotic abbreviated as Unimp/Imp, C/A respectively. (A) Violin plots over raw data points of the mean number of observed OTUs and Shannon Diversity Index for the rarified control and antibiotic-treated groups. 500 rarefactions performed and pairwise comparisons made using 2-sided Tukey's Honest Significant Differences with adjustment for multiple comparisons. (B) Alluvial plot of the mean relative abundance over time by phylum in the rarefied fecal samples from antibiotic-treated and control unimplanted and implanted animals. 500 rarefactions performed. Bands for phyla ordered from most (top) to least (bottom) abundant at each timepoint. (C) Bacteria composition comparison of the gut microbiome using Analysis of Compositions with Bias Correction (ANCOM-BC) between antibiotic and control groups for the unimplanted group after two weeks of treatment, the implanted group after 4 weeks of implantation and treatment, and the implanted group after 12 weeks of implantation and treatment. ANCOM-BC used to perform a 2-sided test for differential abundance (log fold change ≠ 0). Created in BioRender. Capadona, J. (2025) https://BioRender.com/l47u236.

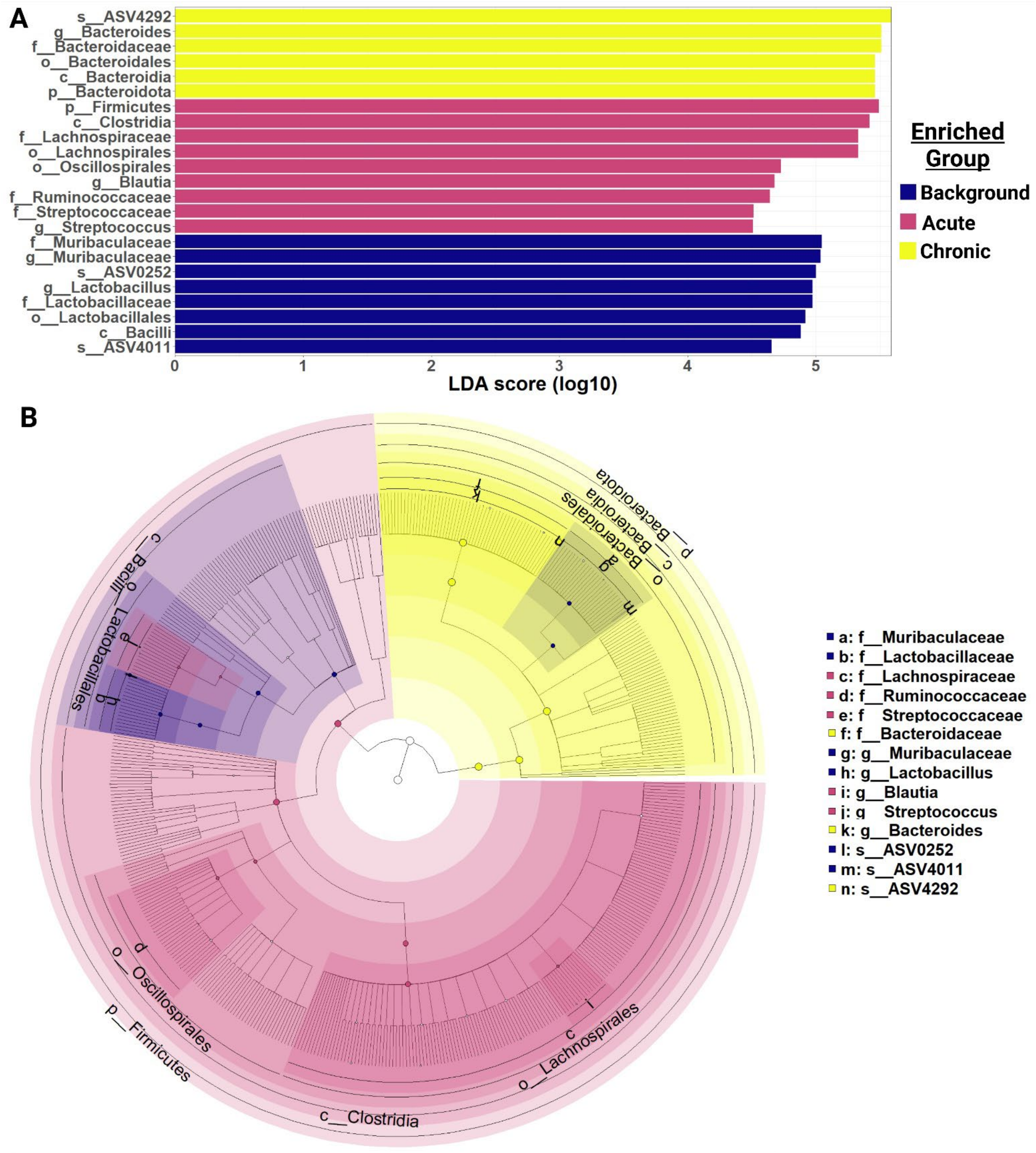


**Supplemental Figure S2: Invasion of the Brain by Microbes from the Phylum Firmicutes Following Microelectrode Implantation.** The relative abundance of microbes varies by implantation status within the control group, with specific taxa enriched in each timepoint, as revealed by Linear discriminant analysis Effect Size (LEfSe) biomarker identification. LEfSe performs a 2-sided non-parametric factorial Kruskal-Wallis (KW) sum-rank test to identify differentially abundant features between groups, pairwise 2-sided unpaired Wilcoxon rank-sum tests to determine biological consistency within subclasses, and estimates effect sizes for each feature with Linear Discriminant Analysis (LDA). Only the most enriched features with axc LDA score greater than $10^{4.5}$ are shown. (A) Bar plot of the log10 LDA scores estimating effect sizes of each differentially abundant feature, where higher values of the indicate a greater degree of enrichment of that feature within that group. (B) A cladogram of the phylogenetic relationship between the enriched taxa from each implantation status. Created in BioRender. Capadona, J. (2025) https://BioRender.com/u64c450.

# 12-Week Antibiotic Baseline to Control

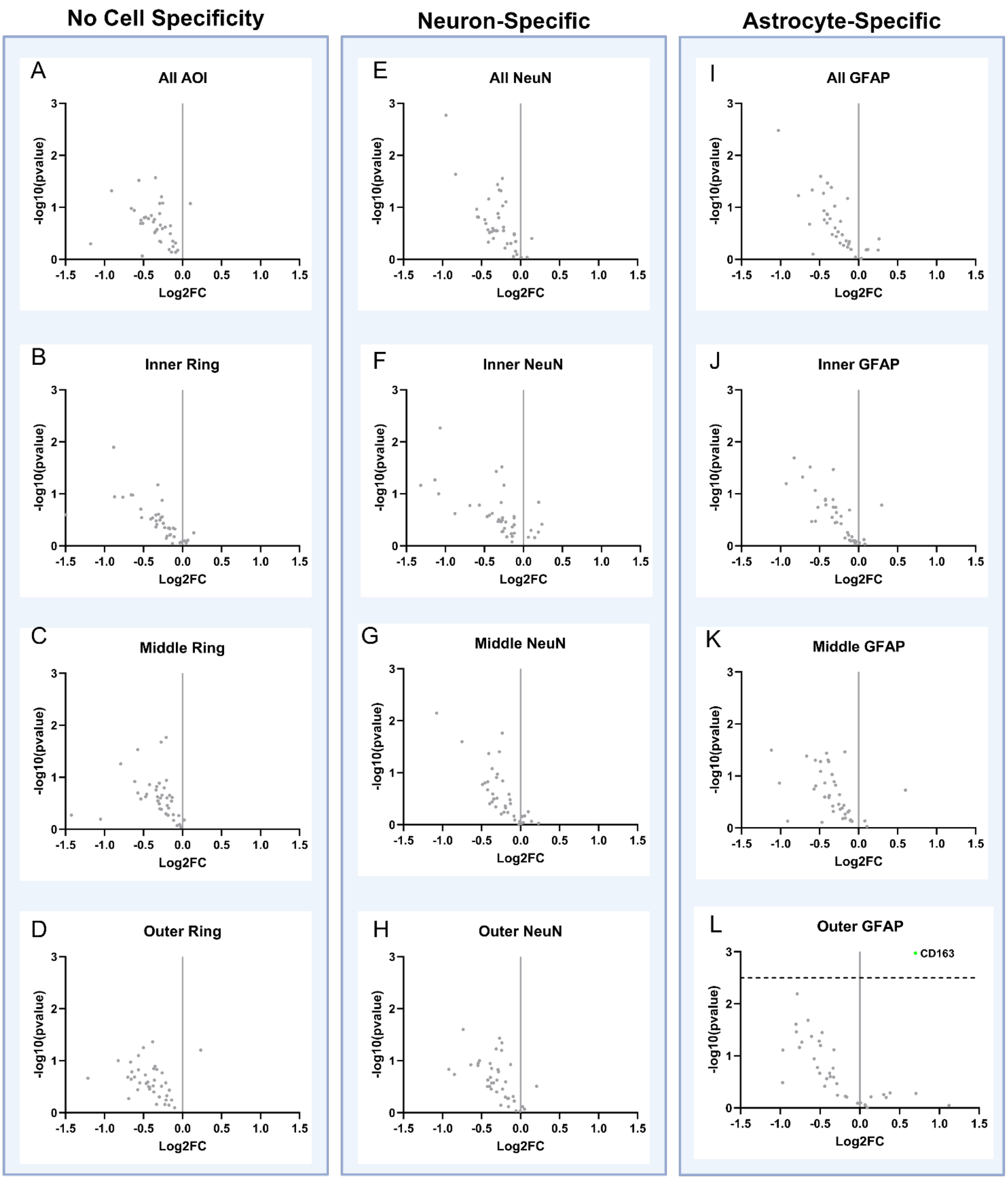


**Supplemental Figure S3: Minimal Proteomic Changes are Observed at 12 Weeks Post-Implantation.** Volcano plots showing neural proteomic panel evaluation of 12-week antibiotic (n = 3 samples) compared to **12**-week control (n = 3 samples) across the entire AOI (within 270 μm from the implant), the inner ring of the AOI (within 0 - 90 μm), the middle ring of the AOI (90 – 180 μm), and the

outer ring (180 - 270 μm) for all cells, all neuron-specific cells (stained using an NeuN antibody), and all astrocyte-specific cells (stained using a GFAP antibody). Proteins with a negative Log2FC indicate downregulation (blue points) in antibiotic compared to control, while a positive Log2FC indicates upregulation (green points) in antibiotic compared to control. Unadjusted p-values are plotted and shown, but all statistical comparisons were done using adjusted p-values. The black dotted line indicates significance ($p_{adjusted} = 0.05$). Each point on the volcano plot indicates a singular protein, with select proteins shown in the text. Comparisons with no cell specificity were made on the (A) entire AOI, (B) inner ring, (C) middle ring, and (D) outer ring. Neuron-specific comparisons were made on the (E) entire AOI, (F) inner ring, (G) middle ring, and (H) outer ring. Astrocyte-specific comparisons were made on the (I) entire AOI, (J) inner ring, (K) middle ring, and (L) outer ring. After normalization, an unpaired t-test were performed across respective groups for comparison. Unadjusted p-values were corrected using the Benjamini-Hochberg false discovery rate method to account for random significance. A few insignificant proteins were excluded from the plots due to high $log_2$(FC) values causing skewing and making visual representation difficult. Created in BioRender. Capadona, J. (2025) https://BioRender.com/p93j360.

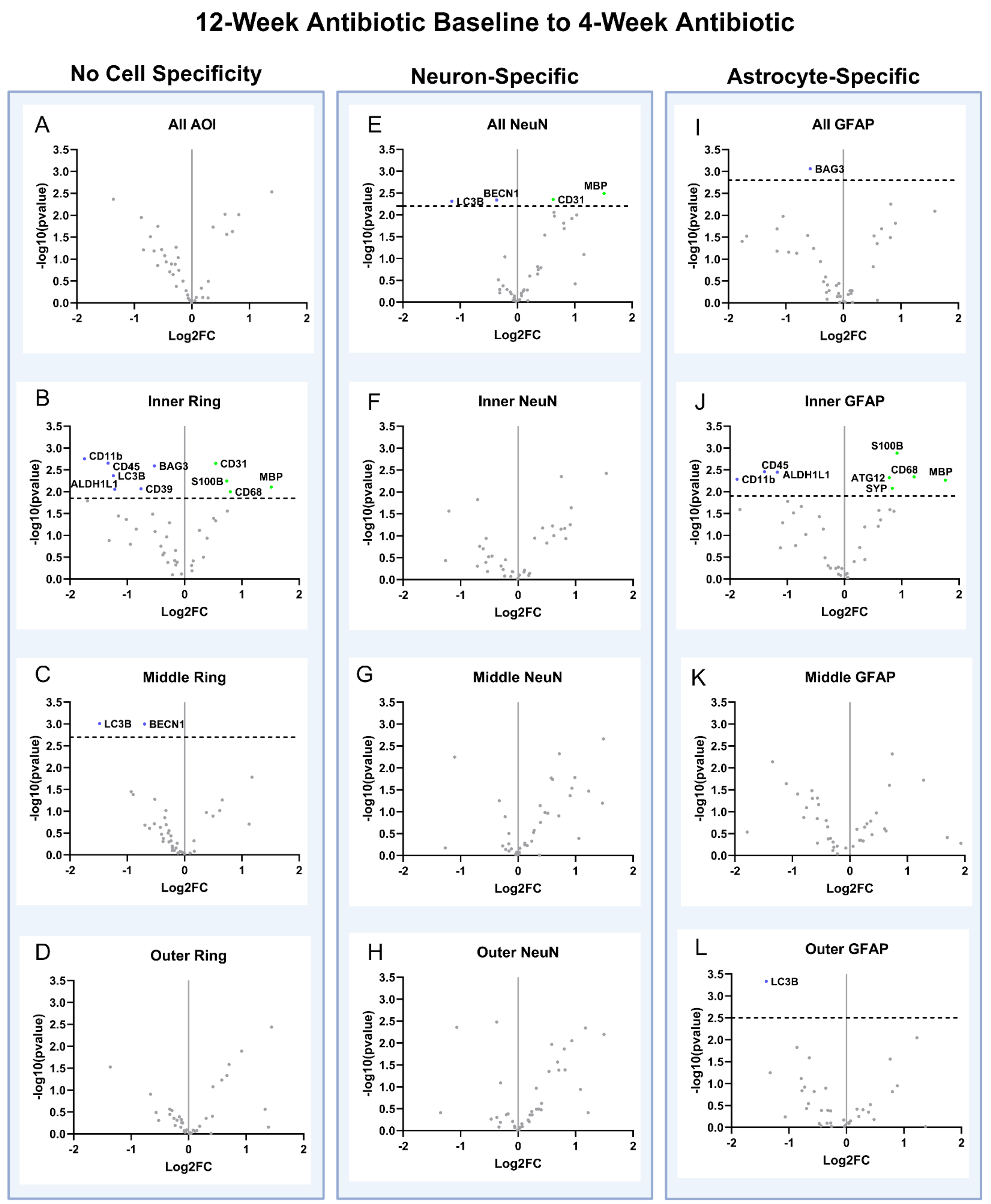


**Supplemental Figure S4: Proteomics of the Antibiotic Group Vary Over Time Within Specific Regions.** Volcano plots showing neural proteomic panel evaluation of 12-week antibiotic (n = 3 samples) compared to 4-week antibiotic (n = 4 samples) across the entire AOI (within 270 μm from the implant), the inner ring of the AOI (within 0 - 90 μm), the middle ring of the AOI (90 – 180 μm), and the outer ring (180 - 270 μm) for all cells, all neuron-specific cells (stained using an NeuN antibody), and all astrocyte-specific cells

(stained using a GFAP antibody). Proteins with a negative Log2FC indicate downregulation (blue points) in antibiotic compared to control, while a positive Log2FC indicates upregulation (green points) in antibiotic compared to control. Unadjusted p-values are plotted and shown, but all statistical comparisons were done using adjusted p-values. The black dotted line indicates significance ($p_{adjusted}$ = 0.05). Each point on the volcano plot indicates a singular protein, with select proteins shown in the text. Comparisons with no cell specificity were made on the (A) entire AOI, (B) inner ring, (C) middle ring, and (D) outer ring. Neuron-specific comparisons were made on the (E) entire AOI, (F) inner ring, (G) middle ring, and (H) outer ring. Astrocyte-specific comparisons were made on the (I) entire AOI, (J) inner ring, (K) middle ring, and (L) outer ring. After normalization, an unpaired t-test were performed across respective groups for comparison. Unadjusted p-values were corrected using the Benjamini-Hochberg false discovery rate method to account for random significance. A few insignificant proteins were excluded from the plots due to high $\log_2$(FC) values causing skewing and making visual representation difficult. Created in BioRender. Capadona, J. (2025) https://BioRender.com/p93j360.

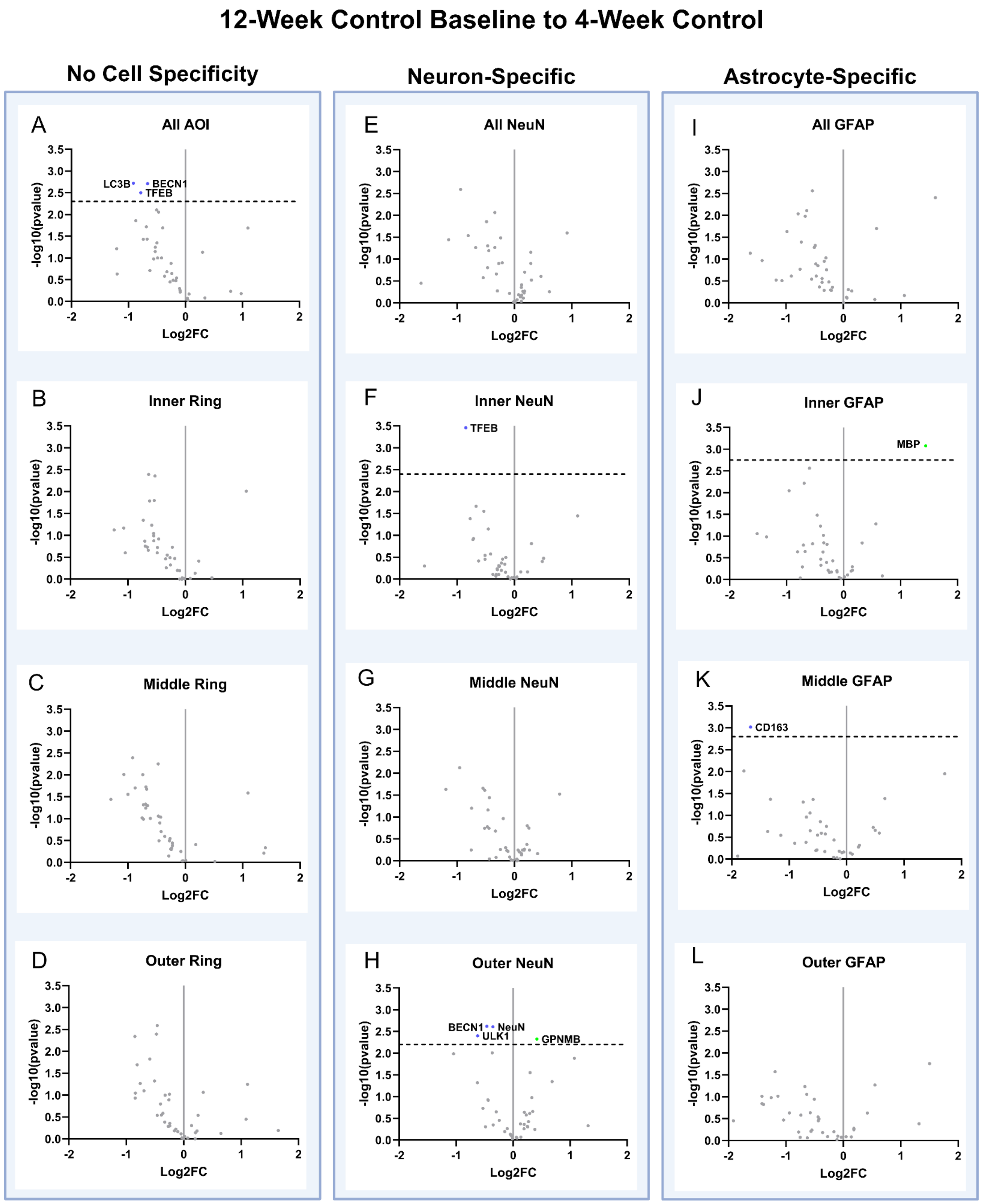


**Supplemental Figure S5: Minor Proteomic Differences Occur In the Control Group Over Time.** Volcano plots showing neural proteomic panel evaluation of 12-week control (n = 3 samples) compared to 4-week control (n = 3 samples) across the entire AOI (within 270 μm from the implant), the inner ring of the AOI (within 0 - 90 μm), the middle ring of the AOI (90 – 180 μm), and the outer

ring (180 - 270 μm) for all cells, all neuron-specific cells (stained using an NeuN antibody), and all astrocyte-specific cells (stained using a GFAP antibody). Proteins with a negative Log2FC indicate downregulation (blue points) in antibiotic compared to control, while a positive Log2FC indicates upregulation (green points) in antibiotic compared to control. Unadjusted p-values are plotted and shown, but all statistical comparisons were done using adjusted p-values. The black dotted line indicates significance ($p_{adjusted} = 0.05$). Each point on the volcano plot indicates a singular protein, with select proteins shown in the text. Comparisons with no cell specificity were made on the (A) entire AOI, (B) inner ring, (C) middle ring, and (D) outer ring. Neuron-specific comparisons were made on the (E) entire AOI, (F) inner ring, (G) middle ring, and (H) outer ring. Astrocyte-specific comparisons were made on the (I) entire AOI, (J) inner ring, (K) middle ring, and (L) outer ring. After normalization, an unpaired t-test were performed across respective groups for comparison. Unadjusted p-values were corrected using the Benjamini-Hochberg false discovery rate method to account for random significance. A few insignificant proteins were excluded from the plots due to high $\log_2$(FC) values causing skewing and making visual representation difficult. Created in BioRender. Capadona, J. (2025) https://BioRender.com/p93j360.

| 12-Week Antibiotic vs 4-Week Antibiotic | | | | | | | | | | | | | | |
|---|---|---|---|---|---|---|---|---|---|---|---|---|---|---|
| Protein Symbol | Protein Name | Category/Function | All AOI | Inner Ring | Middle Ring | Outer Ring | All NeuN | Inner NeuN | Middle NeuN | Outer NeuN | All GFAP | Inner GFAP | Middle GFAP | Outer GFAP |
| ALDH1L1 | aldehyde dehydrogenase 1 family, member L1 | Astrocytes: regulates cell division and growth [1] | | - | | | | | | | | - | | |
| ATG12 | autophagy related 12 | Autophagy: autophagosome protein (KEGG:04140) [2-4] | | | | | | | | | | + | | |
| BAG3 | BCL2-associated athanogene 3 | Autophagy: promotes autophagy through increasing glutamine consumption [5] | | - | | | | | | | - | | | |
| BECN1 | beclin 1, autophagy related | Autophagy: forms protein complex that initiates autophagosome formation [6] | | | - | | - | | | | | | | |
| CD11b | integrin alpha M | Microglia, macrophages, peripheral immune: expressed in activated microglia, macrophages, and dendritic cells; plays a role in cell-cell adhesion during inflammation [7,8] | | - | | | | | | | | - | | |
| CD31 | platelet/endothelial cell adhesion molecule 1 | Endothelial cell marker, mediates BBB permeability, specifically permeability to immune cells [8] | | + | | | + | | | | | | | |
| CD39 | ectonucleoside triphosphate diphosphohydrolase 1 | Myeloid Suppression: Expressed by microglia and involved in both innate and adaptive immunity [9] | | - | | | | | | | | | | |
| CD45 | protein tyrosine phosphatase, receptor type, C | Microglia, macrophages, peripheral immune: Expressed highly in macrophages, less so in microglia, involved in adhesion and T-cell activation [10] | | - | | | | | | | | - | | |
| CD68 | CD68 antigen | Macrophage, Microglia, Myeloid: Highly expressed in macrophages and myeloid cells. General marker for inflammation[11] | | + | | | | | | | | + | | |

| 12-Week Antibiotic vs 4-Week Antibiotic | | | | | | | | | | | | | | |
|---|---|---|---|---|---|---|---|---|---|---|---|---|---|---|
| Protein Symbol | Protein Name | Category/Function | All AOI | Inner Ring | Middle Ring | Outer Ring | All NeuN | Inner NeuN | Middle NeuN | Outer NeuN | All GFAP | Inner GFAP | Middle GFAP | Outer GFAP |
| LC3B | microtubule-associated protein 1 light chain 3 beta | <u>Autophagosome Formation, Protein Sorting:</u> Associated with autophagosome development and maturation[12] | | - | - | | - | | | | | | | - |
| MBP | Myelin basic protein | <u>Neuronal health:</u> Essential for proper myelin membrane formation [13] | | + | | | + | | | | | + | | |
| S100B | S100 protein, beta polypeptide, neural | <u>Antigen, Astrocyte, Inflammation, Melanoma, Tumor:</u> Mainly produced by astrocytes and associated with neurodegenerative diseases as well as neuronal development [14] | | + | | | | | | | | + | | |
| SYP | synaptophysin | <u>Neuronal health:</u> synaptic vesicle protein [15] | | | | | | | | | | + | | |

**Supplemental Table S1: List of Proteins Significantly Impacted in the Antibiotic-Treated Group Over Time.** The list of neural proteomic panels that are differentially expressed between 12-week antibiotic and 4-week antibiotic groups within the entire AOI (All AOI, 270 µm from the implant site), the inner ring (Inner Ring, 90 µm from the implant site), middle ring (Middle Ring, 90 – 180 µm from the implant site), and outer ring (Outer Ring, 180 – 270 µm from the implant site), along with Neuron-specific cells (NeuN) and astrocyte-specific cells (GFAP) for each of those regions. The protein symbol, full name, and function are included in the table. Cells denoted with a “-” indicate downregulation in the 4-week antibiotic group compared to the 4-week control group, while “+” indicates upregulation.

| 12-Week Control vs 4-Week Control | | | | | | | | | | | | | | |
|---|---|---|---|---|---|---|---|---|---|---|---|---|---|---|
| **Protein Symbol** | **Protein Name** | **Category/Function** | **All AOI** | **Inner Ring** | **Middle Ring** | **Outer Ring** | **All NeuN** | **Inner NeuN** | **Middle NeuN** | **Outer NeuN** | **All GFAP** | **Inner GFAP** | **Middle GFAP** | **Outer GFAP** |
| BECN1 | beclin 1, autophagy related | Autophagy: forms protein complex that initiates autophagosome formation [6] | - | | | | | | | - | | | | |
| CD163 | CD163 antigen | M2 Macrophage, Macrophage, Myeloid, Myeloid Suppression: Marker that indicates transition from pro-inflammatory M1 to M2 anti-inflammatory macrophage phenotype[16] | | | | | | | | | | | - | |
| GPNMB | glycoprotein (transmembrane) nmb | Disease-Associated Microglia: Highly expressed in microglia and macrophages during neuroinflammation and neurodegenerative disease states [17] | | | | | | | | + | | | | |
| LC3B | microtubule-associated protein 1 light chain 3 beta | Autophagosome Formation, Protein Sorting: Associated with autophagosome development and maturation[12] | - | | | | | | | | | | | |
| MBP | myelin basic protein | Neuronal health: Essential for proper myelin membrane formation [13] | | | | | | | | | | + | | |
| NeuN | RNA binding protein, fox-1 homolog (C. elegans) 3 | Neuronal health: Binds to DNA in mature neurons, serves as a marker for neuronal nuclei [18] | | | | | | | | - | | | | |
| TFEB | transcription factor EB | Autophagy: promotes transcription of genes that lead to autophagosome formation [19] | - | | | | | - | | | | | | |
| ULK1 | unc-51 like kinase 1 | Autophagy: forms protein complex that initiates autophagosome formation [6] | | | | | | | | - | | | | |

**Supplemental Table S2: List of Proteins Significantly Impacted in the Control Group Over Time.** The list of neural proteomic panels that are differentially expressed between 12-week control and 4-week groups within the entire AOI (All AOI, 270 µm from the implant site), the inner ring (Inner Ring, 90 µm from the implant site), middle ring (Middle Ring, 90 – 180 µm from the implant site), and outer ring (Outer Ring, 180 – 270 µm from the implant site), along with Neuron-specific cells (NeuN) and astrocyte-specific cells (GFAP) for each of those regions. The protein symbol, full name, and function are included in the table. Cells denoted with a "-" indicate downregulation in the 4-week antibiotic group compared to the 4-week control group, while "+" indicates upregulation.

# Ribosomal Protein Subunits

A 4-Week

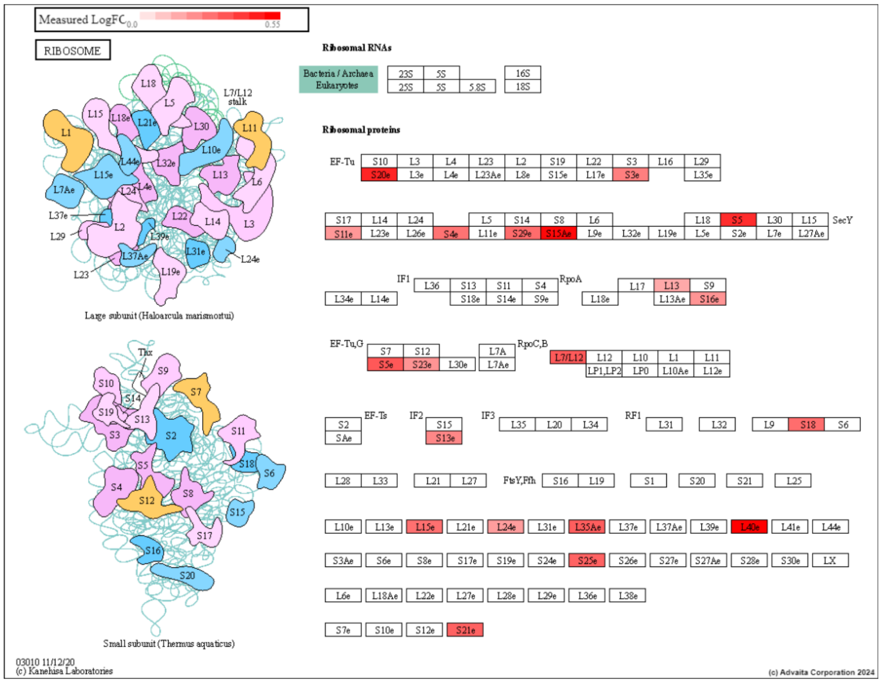


B 12-Week

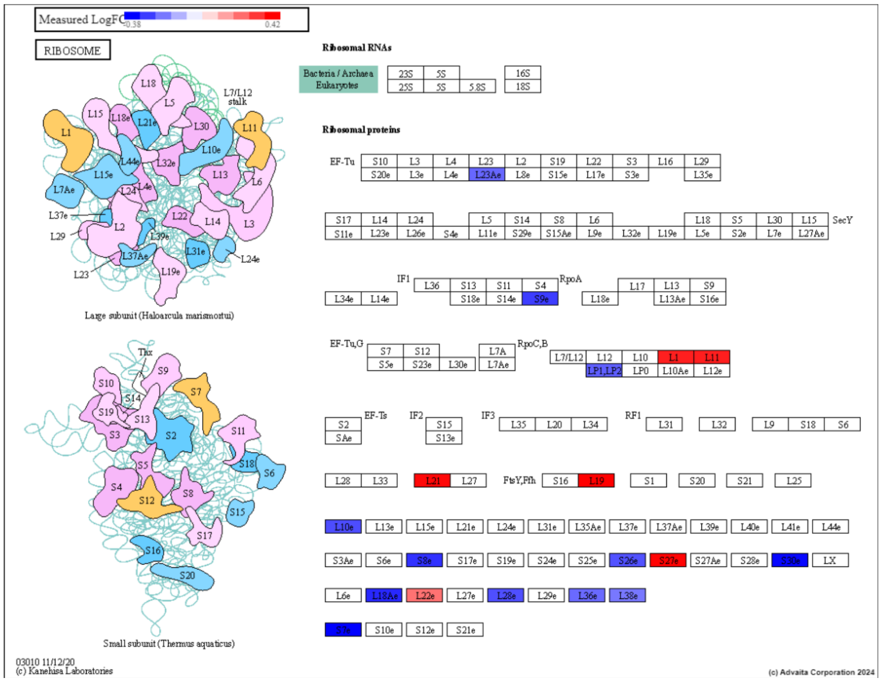


**Supplemental Figure S6: The Ribosomal Subunit Pathway is Significantly Impacted Due to Antibiotic Treatment at 4- and 12-Weeks.** Genes involved in the structure of ribosomal proteins in the large and small subunit (KEGG:03010) are altered due to antibiotic treatment, depending on the time point. (A) At 4 weeks post-implantation, many genes are upregulated compared to control, while (B) many are downregulated **by** 12 weeks post-implantation. Red indicates upregulation in antibiotic animals and blue indicates downregulation in antibiotic animals. Created in BioRender. Capadona, J. (2025) https://BioRender.com/p93j360.

# 4-Week Neurodegeneration Pathway

## A UPS Disruption

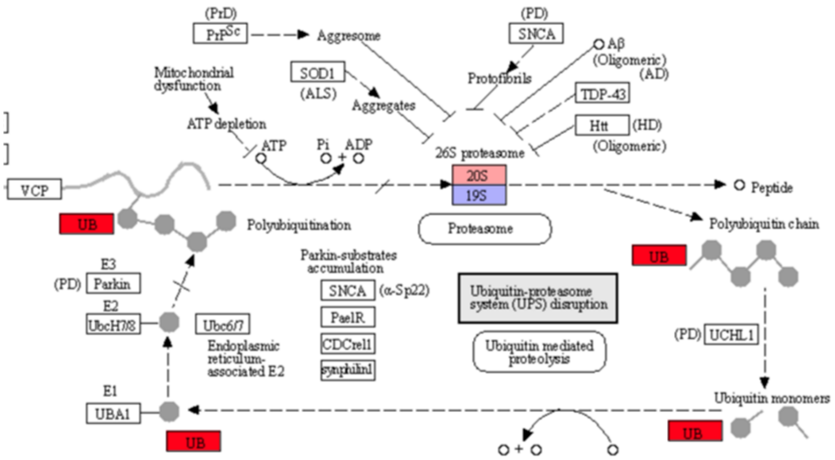


## B Mitochondrial Dysfunction

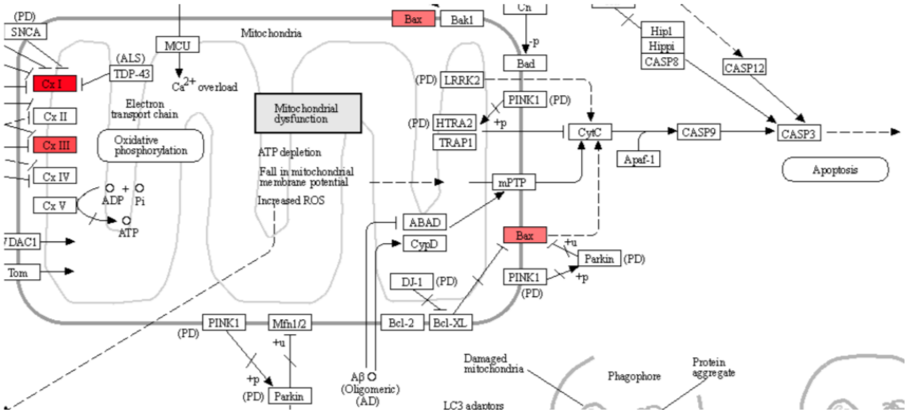


## C Tau Protein Accumulation

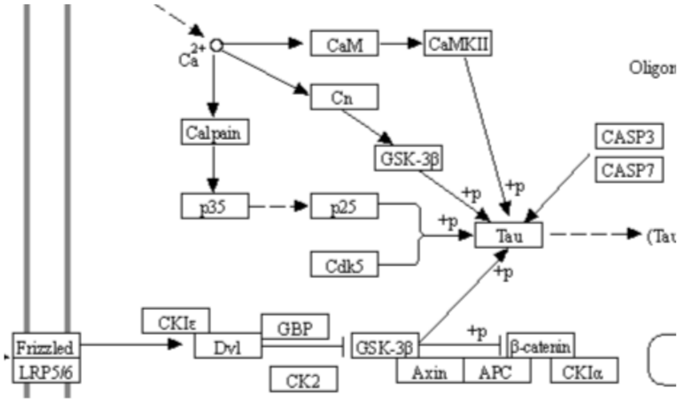


**Supplemental Figure S7: Antibiotic Treatment at 4-Weeks Shows Minor Impacts on Neurodgeneration.** Key pathways associated with the neurodegeneration pathway of diseases (KEGG: 05022) are altered at 4 weeks post-implantation as a result of antibiotic treatment. Notably, (A) UPS disruption and (B) mitochondrial dysfunction are impacted, while (C) taue protein accumulation is unaffected at 4 weeks post-implantation. Created in BioRender. Capadona, J. (2025) https://BioRender.com/p93j360.

# 12-Week Neurodegeneration Pathway

## A UPS Disruption

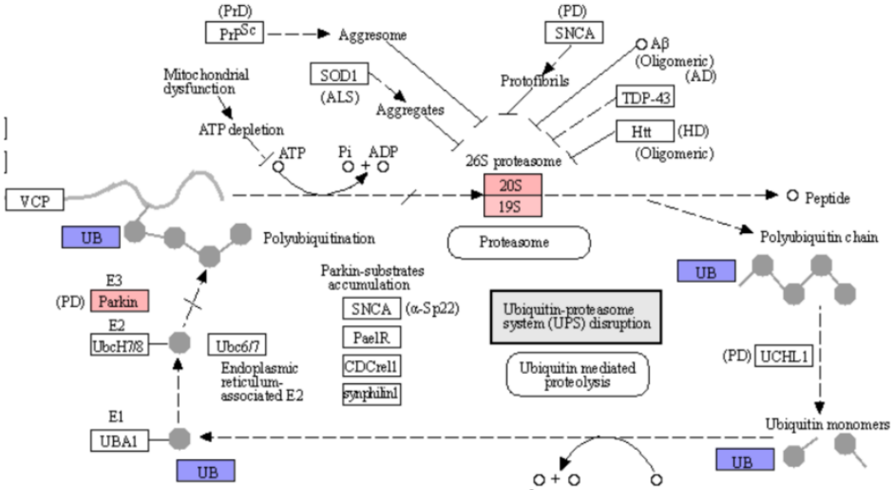


## B Mitochondrial Dysfunction

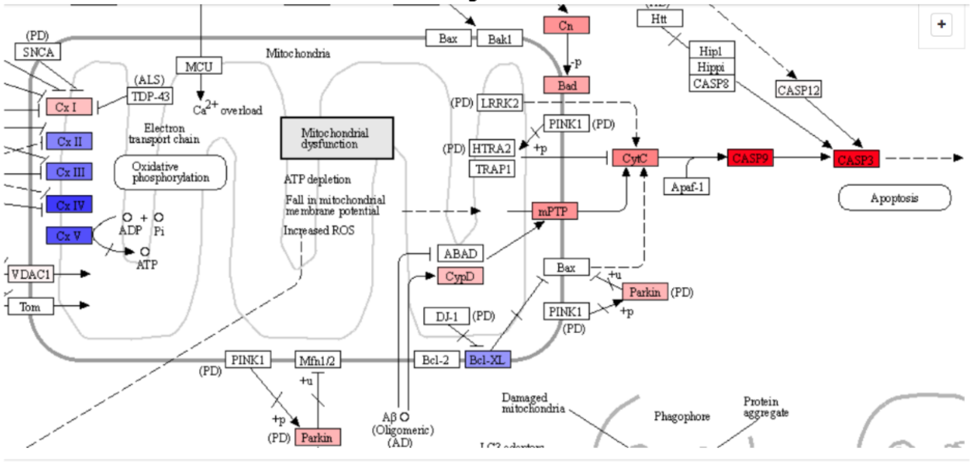


## C Tau Protein Accumulation

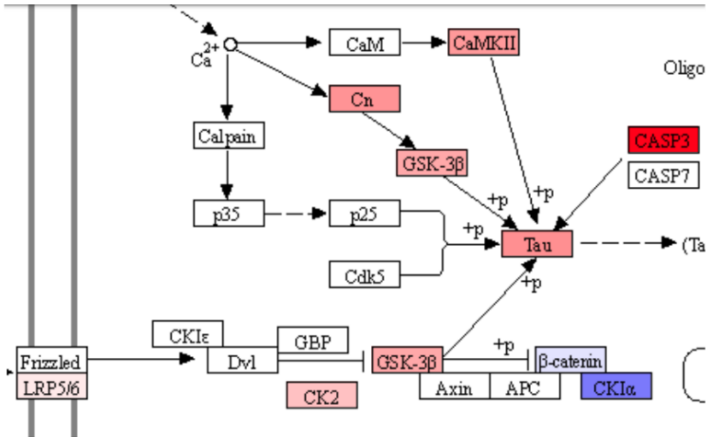


**Supplemental Figure S8: Antibiotic Treatment Significantly Impacts Pathways of Neurodegeneration at 12-Weeks Post-Implantation.** Key pathways associated with the neurodegeneration pathway of diseases (KEGG: 05022) are altered as a result of antibiotic treatment at 12 weeks post-implantation. Notably, (A) UPS disruption, (B) mitochondrial dysfunction, and (C) tau protein accumulation are impacted. Created in BioRender. Capadona, J. (2025) https://BioRender.com/p93j360.

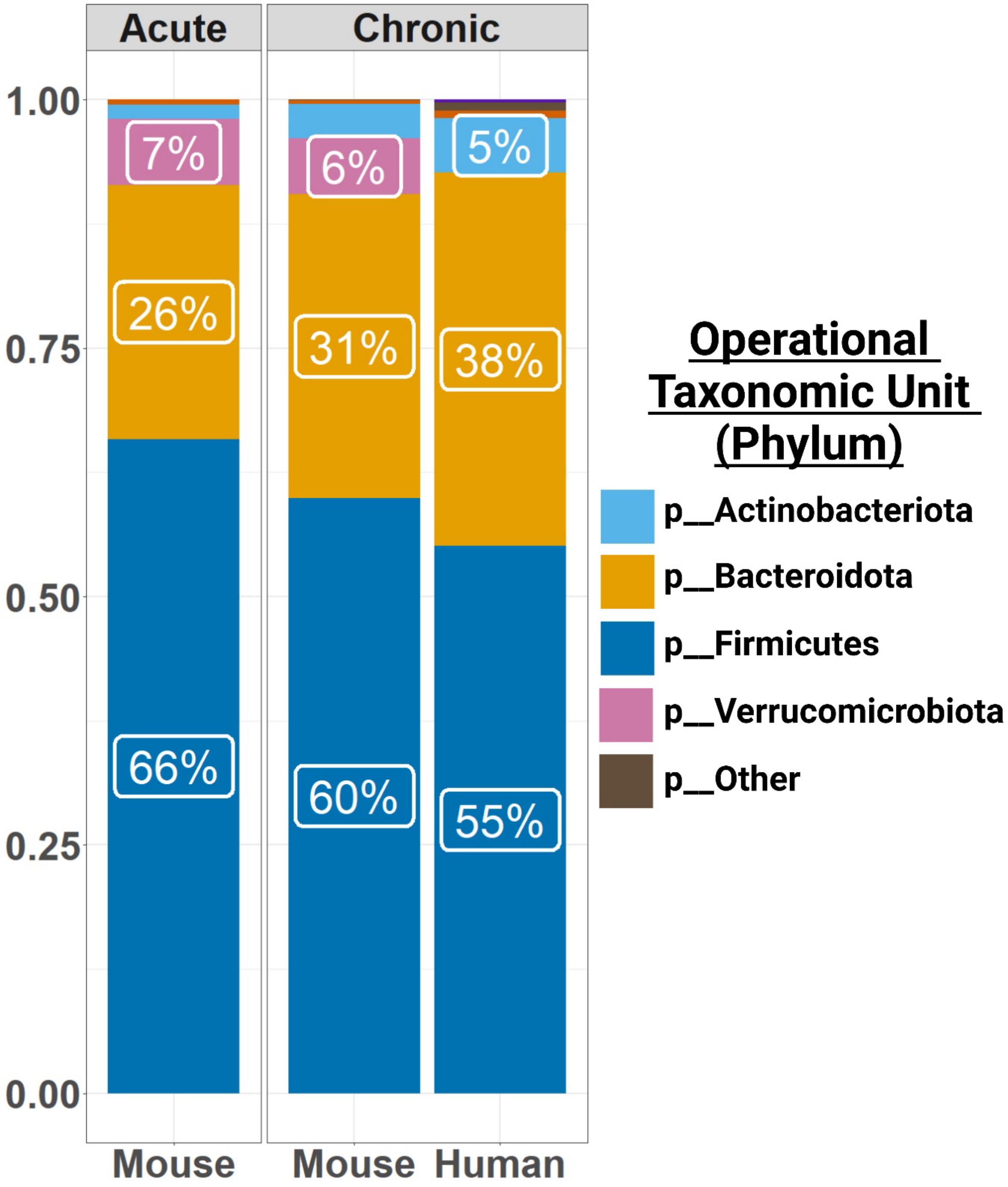


**Supplemental Figure S9. Cross-species microbiome analysis for intracortical microelectrode implanted mice and a human subject.** Bar plots of the relative abundance of gut bacterial sequences in a human subject versus the mean relative abundance of gut bacterial sequences in implanted mice from 500 rarefactions by phylum and stage. Over 90% of the relative abundance of gut-derived bacteria is consistent at the phylum level in our microelectrode-implanted mice and human participant. Created in BioRender. Capadona, J. (2025) https://BioRender.com/k06h230.

## References Cited


1 Yang, Y. *et al.* Molecular comparison of GLT1+ and ALDH1L1+ astrocytes in vivo in astroglial reporter mice. *Glia* **59**, 200-207, doi:10.1002/glia.21089 (2011).

2 Kanehisa, M. Toward understanding the origin and evolution of cellular organisms. *Protein Sci* **28**, 1947-1951, doi:10.1002/pro.3715 (2019).

3 Kanehisa, M., Furumichi, M., Sato, Y., Kawashima, M. & Ishiguro-Watanabe, M. KEGG for taxonomy-based analysis of pathways and genomes. *Nucleic Acids Res* **51**, D587-D592, doi:10.1093/nar/gkac963 (2023).

4 Kanehisa, M. & Goto, S. KEGG: kyoto encyclopedia of genes and genomes. *Nucleic Acids Res* **28**, 27-30, doi:10.1093/nar/28.1.27 (2000).

5 Zhao, S. *et al.* BAG3 promotes autophagy and glutaminolysis via stabilizing glutaminase. *Cell Death Dis* **10**, 284, doi:10.1038/s41419-019-1504-6 (2019).

6 Menon, M. B. & Dhamija, S. Beclin 1 Phosphorylation - at the Center of Autophagy Regulation. *Front Cell Dev Biol* **6**, 137, doi:10.3389/fcell.2018.00137 (2018).

7 Jurga, A. M., Paleczna, M. & Kuter, K. Z. Overview of General and Discriminating Markers of Differential Microglia Phenotypes. *Front Cell Neurosci* **14**, 198, doi:10.3389/fncel.2020.00198 (2020).

8 Sun, X. & Kaufman, P. D. Ki-67: more than a proliferation marker. *Chromosoma* **127**, 175-186, doi:10.1007/s00412-018-0659-8 (2018).

9 Moesta, A. K., Li, X. Y. & Smyth, M. J. Targeting CD39 in cancer. *Nat Rev Immunol* **20**, 739-755, doi:10.1038/s41577-020-0376-4 (2020).

10 Altin, J. G. & Sloan, E. K. The role of CD45 and CD45-associated molecules in T cell activation. *Immunol Cell Biol* **75**, 430-445, doi:10.1038/icb.1997.68 (1997).

11 Chistiakov, D. A., Killingsworth, M. C., Myasoedova, V. A., Orekhov, A. N. & Bobryshev, Y. V. CD68/macrosialin: not just a histochemical marker. *Lab Invest* **97**, 4-13, doi:10.1038/labinvest.2016.116 (2017).

12 Schaaf, M. B., Keulers, T. G., Vooijs, M. A. & Rouschop, K. M. LC3/GABARAP family proteins: autophagy-(un)related functions. *FASEB J* **30**, 3961-3978, doi:10.1096/fj.201600698R (2016).

13 Kister, A. & Kister, I. Overview of myelin, major myelin lipids, and myelin-associated proteins. *Front Chem* **10**, 1041961, doi:10.3389/fchem.2022.1041961 (2022).

14 Rothermundt, M., Peters, M., Prehn, J. H. & Arolt, V. S100B in brain damage and neurodegeneration. *Microsc Res Tech* **60**, 614-632, doi:10.1002/jemt.10303 (2003).

15 Wiedenmann, B., Franke, W. W., Kuhn, C., Moll, R. & Gould, V. E. Synaptophysin: a marker protein for neuroendocrine cells and neoplasms. *Proc Natl Acad Sci U S A* **83**, 3500-3504, doi:10.1073/pnas.83.10.3500 (1986).

16 Alvarado-Vazquez, P. A. *et al.* Macrophage-specific nanotechnology-driven CD163 overexpression in human macrophages results in an M2 phenotype under inflammatory conditions. *Immunobiology* **222**, 900-912, doi:10.1016/j.imbio.2017.05.011 (2017).

17 Saade, M., Araujo de Souza, G., Scavone, C. & Kinoshita, P. F. The Role of GPNMB in Inflammation. *Front Immunol* **12**, 674739, doi:10.3389/fimmu.2021.674739 (2021).

18 Wolf, H. K. *et al.* NeuN: a useful neuronal marker for diagnostic histopathology. *J Histochem Cytochem* **44**, 1167-1171, doi:10.1177/44.10.8813082 (1996).

19 Huang, J. *et al.* Exercise activates lysosomal function in the brain through AMPK-SIRT1-TFEB pathway. *CNS Neurosci Ther* **25**, 796-807, doi:10.1111/cns.13114 (2019).
