# Supplementary material for: Bacteria invade the brain following intracortical microelectrode implantation, inducing gut-brain axis disruption and contributing to reduced microelectrode performance
Source: Nat Commun. 2025 Feb 20;16:1829. doi: 10.1038/s41467-025-56979-4 (PMC11842729; doi:10.1038/s41467-025-56979-4)
Supplement: Supplementary file 3 — Reporting Summary [file 41467_2025_56979_MOESM3_ESM.pdf]

nature portfolio

Corresponding author(s): Capadona, Jeffrey R

Last updated by author(s): Jan 3, 2025

Reporting Summary

Nature Portfolio wishes to improve the reproducibility of the work that we publish. This form provides structure for consistency and transparency in reporting. For further information on Nature Portfolio policies, see our Editorial Policies and the Editorial Policy Checklist.

Statistics

For all statistical analyses, confirm that the following items are present in the figure legend, table legend, main text, or Methods section.

n/a	Confirmed	
☐	☒	The exact sample size (*n*) for each experimental group/condition, given as a discrete number and unit of measurement
☐	☒	A statement on whether measurements were taken from distinct samples or whether the same sample was measured repeatedly
☐	☒	The statistical test(s) used AND whether they are one- or two-sided<br>*Only common tests should be described solely by name; describe more complex techniques in the Methods section.*
☐	☒	A description of all covariates tested
☐	☒	A description of any assumptions or corrections, such as tests of normality and adjustment for multiple comparisons
☐	☒	A full description of the statistical parameters including central tendency (e.g. means) or other basic estimates (e.g. regression coefficient) AND variation (e.g. standard deviation) or associated estimates of uncertainty (e.g. confidence intervals)
☐	☒	For null hypothesis testing, the test statistic (e.g. *F*, *t*, *r*) with confidence intervals, effect sizes, degrees of freedom and *P* value noted<br>*Give P values as exact values whenever suitable.*
☐	☒	For Bayesian analysis, information on the choice of priors and Markov chain Monte Carlo settings
☒	☐	For hierarchical and complex designs, identification of the appropriate level for tests and full reporting of outcomes
☐	☒	Estimates of effect sizes (e.g. Cohen's *d*, Pearson's *r*), indicating how they were calculated

Our web collection on statistics for biologists contains articles on many of the points above.

Software and code

Policy information about availability of computer code

Data collection	No proprietary codes were developed for data collection. Data collection software include Plexon Offline Sorter, Synapse recording software, Illumina MiSeq system software, NanoString GeoMx and nCounter software, and NanoString's NGS pipeline software.
Data analysis	No proprietary codes were developed for this data analysis. Software used for analysis include R Studio 2022.7.1+554, MATLAB R2021a, iPathways software suite, GraphPad Prism Plus 10, and QIIME2 v2023.5. All custom codes are available within the studies OSF repository.

For manuscripts utilizing custom algorithms or software that are central to the research but not yet described in published literature, software must be made available to editors and reviewers. We strongly encourage code deposition in a community repository (e.g. GitHub). See the Nature Portfolio guidelines for submitting code & software for further information.

Data

Policy information about availability of data

All manuscripts must include a data availability statement. This statement should provide the following information, where applicable:

- Accession codes, unique identifiers, or web links for publicly available datasets
- A description of any restrictions on data availability
- For clinical datasets or third party data, please ensure that the statement adheres to our policy

Raw and processed data can be found under our OSF repository located at DOI 10.17605/OSF.IO/JKG27.

Research involving human participants, their data, or biological material

Policy information about studies with human participants or human data. See also policy information about sex, gender (identity/presentation), and sexual orientation and race, ethnicity and racism.

Reporting on sex and gender	N/A
Reporting on race, ethnicity, or other socially relevant groupings	N/A
Population characteristics	N/A
Recruitment	N/A
Ethics oversight	N/A

Note that full information on the approval of the study protocol must also be provided in the manuscript.

Field-specific reporting

Please select the one below that is the best fit for your research. If you are not sure, read the appropriate sections before making your selection.

☒ Life sciences ☐ Behavioural & social sciences ☐ Ecological, evolutionary & environmental sciences

For a reference copy of the document with all sections, see nature.com/documents/nr-reporting-summary-flat.pdf

Life sciences study design

All studies must disclose on these points even when the disclosure is negative.

Sample size

Sample size for the week-by-week "Proportion of Active Electrodes" was determined by summing the total number of electrodes multiplied by the number of animals in each group on a week-by-week basis. The sample size for acute, sub-chronic, and chronic "Proportion of Active Electrodes" was determined by summing the total number of electrodes multiplied by the number of weeks in each phase and the number of animals in each group. The sample size for the additional recording metrics was calculated by averaging the respective recording metric on a per-channel basis, and then summing up all unique channels across all animals within the same group and time point (e.g. if channel 1 of antibiotic animal 1 records an SNR on weeks 1, 2, and 3, then those values were averaged into a singular SNR value for channel 1 during the acute phase of antibiotic animal 1 to be used in further analysis).

For transcriptomics and proteomics a sample size of n = 3 or 4 was largely based on the extremely high cost of the experiment in combination with running a power analysis after our initial samples and finding they were significant. This was the same rationale behind the sample size chosen for our 16S experiments. Due to higher variance in data, the power analysis required a larger sample size,m which is why samples were taken from most implanted animals to be used in 16S analysis.

Data exclusions

In brain tissues and fecal samples, there is a significant inverse relationship between the relative abundance of contaminant DNA and a sample's read count, which aligns with the statistical assumption and validates the exclusion of these sequences from further analysis. Host gDNA was the primary contaminant in brain tissue samples, while technical contaminants were more abundant in the fecal samples. The abundance of sequences categorized as contaminating microbial DNA was negatively correlated with total read count in fecal samples, but it was not correlated with total read count in brain tissues. Extraction reagent blanks were not used, so there may have been an incomplete removal of technical contaminants from the lowest read count samples. In contrast, the relative abundance of the remaining sequences increased with increasing sample read count in both brain tissues and fecal samples, suggesting that these remaining reads are not contaminants and describe host-derived bacterial DNA.

Replication

Our hypothesis was to test whether changing the gut microbiome can impact neural recording performance and brain health after implantation. We performed conceptual replication by testing this hypothesis in four different experiments: 16S evaluation of the brain after implantation and treatment, neural recording performance, proteomic evaluation around the implant site, and transcriptomic evaluation around the implant site. There was no direct replication or systematic replication performed in this study. However, future studies will be utilized to perform both direct and systematic replication of these results.

Randomization

The brains selected for transcriptomics and proteomics analysis were randomly selected from the total available brain samples from the study. For 16S and recording analysis, all brains were used, so there was no need to randomize selection of samples.

Blinding

During neurophysiological recording data collection, the data collector was blinded to sample groups to avoid any inherent bias. Only the researcher responsible for providing antibiotic treatment was knowledgeable of the groups. This also applies to brain extraction during perfusion and sample collection for subsequent 16S, proteomics, and transcriptomic analysis. Proteomic and transcriptomic preparation and analysis was not blinded, as the researchers needed to confirm that there were 3 or 4 samples available per group to perform analysis on.

Reporting for specific materials, systems and methods

We require information from authors about some types of materials, experimental systems and methods used in many studies. Here, indicate whether each material, system or method listed is relevant to your study. If you are not sure if a list item applies to your research, read the appropriate section before selecting a response.

Materials & experimental systems

n/a	Involved in the study
☐	☒ Antibodies
☒	☐ Eukaryotic cell lines
☒	☐ Palaeontology and archaeology
☐	☒ Animals and other organisms
☐	☒ Clinical data
☒	☐ Dual use research of concern
☒	☐ Plants

Methods

n/a	Involved in the study
☒	☐ ChIP-seq
☒	☐ Flow cytometry
☒	☐ MRI-based neuroimaging

Antibodies

Antibodies used	Neuron Stain 1:100 anti-NeuN, Alexa Fluor® 647 EPR12763, Item Number: ab190565<br>Astrocyte Stain 1:40 anti-GFAP, Alexa Fluor® 532 GA-5, Item Number: NBP2-33184AF532<br>Proprietary Nanostring Proteomic Antibodies 1:25 Item Number: 121300120, Item Number: 121300125, Item Number: 121300124<br>Nuclear Stain 1:10 Syto13, NanoString Technologies, #121300303
Validation	Extensive previous work has been done using NeuN and GFAP antibodies for staining. Nanostring protocols have indicated validated dilutions for their proprietary stains and SYTO13 stain utilized for proteomics and transcriptomics.

Animals and other research organisms

Policy information about studies involving animals; ARRIVE guidelines recommended for reporting animal research, and Sex and Gender in Research

Laboratory animals	All mice used were C57BL/6 mice obtained from Jackson Labs and aged between 8-10 weeks.
Wild animals	None
Reporting on sex	All animal species were male, so there were no sex-specific analyses performed.
Field-collected samples	N/A
Ethics oversight	All procedures and animal care protocols were performed in compliance with Case Western Reserve University's Institutional Animal Care and Use Committee (IACUC) approved protocol.

Note that full information on the approval of the study protocol must also be provided in the manuscript.

Clinical data

Policy information about clinical studies

All manuscripts should comply with the ICMJE guidelines for publication of clinical research and a completed CONSORT checklist must be included with all submissions.

Clinical trial registration	#NCT03898804
Study protocol	We are not at liberty to share the full clinical trial protocol because it is from an ongoing study.
Data collection	Human fecal matter was collected under an approved IRB protocol at University Hospitals Cleveland Medical Center in collaboration with the Reconnecting the Hand and Arm to the Brain (ReHAB) clinical trial (clincaltrials.gov #NCT03898804). At the time of sample collection, the study participant (coded RP1) was a 29-year-old male who had suffered spinal cord injury (C3/C4, AIS B), resulting in tetraplegia (motor-complete, sensory-incomplete), 8 years prior. His participation in the ReHAB pilot clinical trial has been previously reported. Briefly, RP1 received six 64-channel (8x8) Utah intracortical microelectrode arrays (Blackrock Microsystems, Salt Lake City, UT) implanted into various sensorimotor cortices for the purpose of restoring cortically-controlled movements of his paralyzed arm and hand, reanimated by functional electrical stimulation through composite flat interface nerve cuff electrodes. RP1 received the cortical implants 2 years and 6 months prior to fecal sample collection. Fecal matter was collected using standard procedures and transported in sterile and sealed containers, packaged in dry ice, for subsequent analysis. Samples were stored in a -80°C freezer until processing until sequencing.

Outcomes	As this data is from an ongoing clinical study, and we are only using a small portion for supplemental information, we are not at liberty to share the outcomes associated with the clinical trial going on.

Plants

Seed stocks	N/A
Novel plant genotypes	N/A
Authentication	N/A
